# Modeling electronic and optical properties of III–V quantum dots—selected recent developments

**DOI:** 10.1038/s41377-021-00700-9

**Published:** 2022-01-17

**Authors:** Alexander Mittelstädt, Andrei Schliwa, Petr Klenovský

**Affiliations:** 1 Institute for Solid State Physics, Technical University of Berlin, Hardenbergstrasse 36, D-10623 Berlin, Germany; 2 Department of Condensed Matter Physics, Faculty of Science, Masaryk University, Kotlářská 267/2, 61137 Brno, Czech Republic; 3 Czech Metrology Institute, Okružní 31, 63800 Brno, Czech Republic

**Keywords:** Quantum cascade lasers, Photonic devices

## Abstract

Electronic properties of selected quantum dot (QD) systems are surveyed based on the multi-band **k·p** method, which we benchmark by direct comparison to the empirical tight-binding algorithm, and we also discuss the newly developed “linear combination of quantum dot orbitals” method. Furthermore, we focus on two major complexes: First, the role of antimony incorporation in InGaAs/GaAs submonolayer ﻿QDs and In_1−*x*_Ga_*x*_ As_*y*_Sb_1−*y*_/GaP QDs, and second, the theory of QD-based quantum cascade lasers and the related prospect of room temperature lasing.

## Introduction

Semiconductor quantum dots (QDs) are a customized synthetic equivalent to atoms that have found uses in a wide range of modern semiconductor devices^1^. Nanostructures based solely on III–V-system material compounds provide already a wide spectrum of electronic and optical characteristics. This article will demonstrate their immense potential^2^ and tunability^3–5^ by concentrating on the electronic structure of three unique keystone systems of today’s research: (i) Sb-InAs/GaAs submonolayer QDs, (ii) In_1−*x*_Ga_*x*_ As_*y*_Sb_1−*y*_/GaP QDs and (iii) QD based quantum cascade lasers.

(i) For the last 20 years^1,6^, InAs/GaAs QDs have been the focus of comprehensive research, leading to the development of quantum dot lasers^7^ and single-photon emitters^2,8^. To increase QD density and improve carrier dynamics, submonolayer quantum dots (SML QDs) were developed as an complementary approach for QD formation^9,10^: Deposition of fewer than one monolayer (ML) of InAs on GaAs, followed by a small spacer layer of GaAs, is repeated multiple times to form a submonolayer stack. In this paper, we discuss how Sb, as a further atomic species, affects the optical and electronic properties of InAs/GaAs submonolayer stacks.
(ii) One of the major challenges of future photonics^11^ is the monolithic integration of III-V compounds with silicon technology: GaP is the III-V binary compound with the nearest lattice constant to silicon (0.37 percent lattice mismatch at room temperature) and—despite being an indirect semiconductor—works well as a matrix for the In_1−*x*_Ga_*x*_As_*y*_Sb_1−*y*_ material combination, which is discussed here for its potential as an optoelectronic material or its suitability as a storage element. To this purpose, we use an extended version of our previous model that includes X- and L-point states now.
(iii) Suris suggested quantum cascade lasers (QCL) having an active zone made of quantum dot (QD) chains in 1996^12^. Despite their enormous prospective, their realization has so far been delayed mostly by a lack of fast and accurate electronic structure modeling tools for large ensembles of closely aligned QDs. For this reason, we have established the “linear combination of quantum dot orbitals” (LCQO) approach and used it to the construction of a terahertz QD-QCL which lase at room temperature.

Due to the necessity for both fast and precise electronic structure calculations, 3D QD models that go beyond effective mass theory^13^ were developed, where simulations based on eight-band **k**·**p** theory^14,15^ are among the most transparent ones.

The attractiveness of the multi-band **k**·**p** model stems from the appealing balance of accuracy, computation speed, and clarity of the physics and parameters employed. For any geometry^16^ and composition, all important aspects such like confinement, strain, piezo- and pyroelectricity, band-coupling, and -splitting may be faithfully addressed. This brings up the possibility of using the model for inverse bandstructure modeling^16^ and inverse design^17^.

## Method of calculation

**Fig. 1:**
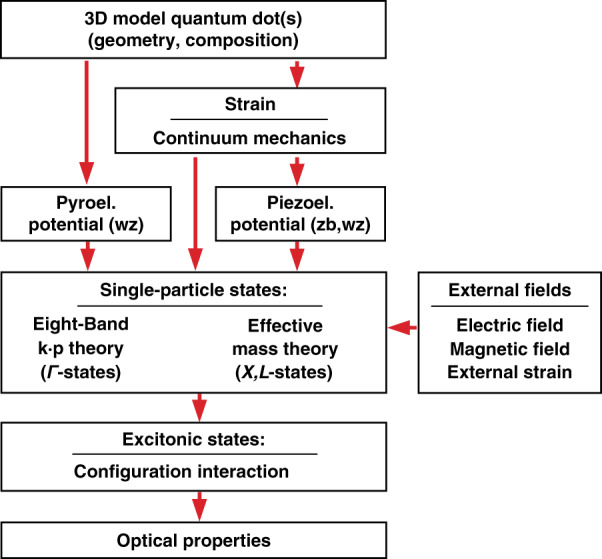
Schematics of the general modeling procedure

The modeling approach used in this paper is depicted schematically in Fig. 1. It begins with the definition of a 3D QD morphology and continues with strain, piezoelectricity, and pyroelectricity calculations. The strain and polarization fields that arise are subsequently passed into the 8-band **k**·**p** Hamiltonian. The single-particle states are calculated by solving the Schrödinger equation. The configuration interaction method, which is based on either the original single-particle states or Hartree-Fock modified states^18^, can be used to account for Coulomb interaction. Finally, optical parameters including absorption spectra, capture cross-sections, and lifetimes can be determined. To keep the reasoning focused, we do not discuss excitonic effects in this work.

### Calculation of strain

Because the strain impact on confinement is equivalent to the band offset caused by chemical composition variations at heterojunctions, the energies and wavefunctions are substantially influenced by the associated strain distribution. Several studies^15,19^ have looked at the suitability of the continuum elasticity model utilized to generate the strain distribution in our **k**·**p** model: The continuum elasticity model, according to Stier et al.^15^, provides more reliable outcomes for QDs compared to Keatings valence force field (VFF) model in its linearized Kane version, where large parts of the strain distribution differences are ascribed to an incorrect elastic constant value C_44_ in the VFF model, rather than its atomistic nature. Zunger et al.^20^ later proposed the G-VFF model, a modified version of the VFF model that correctly incorporates C_44_. For the lattice relaxation, more recent versions of the tight-binding model use Stillinger-Webber-^21^ and Abel-Tersoff^22^ force field potentials.

Because the **k**·**p** model includes a restricted number of parameters only to incorporate strain, it is not receptive to the entire information provided by an atomistic strain model; hence, the continuum model remains the best choice in the context of our electronic structure model.

### Piezoelectricity

The creation of electric polarization by applying stress to a crystal that lacks a center of symmetry is known as piezoelectricity^23^. The zincblende structure is the most basic example of this kind of lattice, with one constant, e_14_, for the linear and three constants, B_114_, B_124_, and B_156_ for the quadratic^24^ terms, yielding:

$$\begin{array}{ll}&P_1 = 2\,e_{14}\left( {{\it{\epsilon }}_{yz}\,{\it{\epsilon }}_{xz}\,{\it{\epsilon }}_{xy}} \right)\\ &P_2 = 2\,B_{114}\left( {{\it{\epsilon }}_{xx}{\it{\epsilon }}_{yz}\,{\it{\epsilon }}_{yy}{\it{\epsilon }}_{xz}\,{\it{\epsilon }}_{zz}{\it{\epsilon }}_{xy}} \right) \\ &\qquad\, + \,2\,B_{124}\left( {{\it{\epsilon }}_{yz}\left( {{\it{\epsilon }}_{yy} + {\it{\epsilon }}_{zz}} \right)\,{\it{\epsilon }}_{xz}\left( {{\it{\epsilon }}_{zz} + {\it{\epsilon }}_{xx}} \right){\it{\epsilon }}_{xy}\left( {{\it{\epsilon }}_{xx} + {\it{\epsilon }}_{yy}} \right)} \right)\\ &\qquad\, + \,2\,B_{156}\left( {{\it{\epsilon }}_{xz}{\it{\epsilon }}_{xy}\,{\it{\epsilon }}_{yz}{\it{\epsilon }}_{xy}\,{\it{\epsilon }}_{yz}{\it{\epsilon }}_{xz}} \right)\end{array}$$

The resulting polarization **P**_zb_ then comprises of two components

$$P_{zb} = P_1 + P_2$$

Bester et al.^24^ have highlighted the relevance of the second-order term **P**_2_ for InGaAs/GaAs(111) quantum wells (QW) and quantum dots (QDs). They discovered that the linear and quadratic coefficients have opposing impact on the potential for QWs, and that the quadratic term prevails for large strain.

The problem, however, is much deeper for InGaAs/GaAs QDs because, further to the enormous strain, their three-dimensional morphology is involved: A quadrupole-like potential is generated by the linear term, which reduces a QD’s structural C_4v_–or C_∞v_ -symmetry to C_2v_^6,25^. Bester et al.^26^ investigated the effect of the quadratic terms on circular-shaped QDs and discovered that they compensate the first-order field inside the QD, resulting in a potential-free QD. Later, the research was expanded to include more realistic morphologies such as truncated pyramids and uneven alloyings^27–29^.

**Fig. 2:**
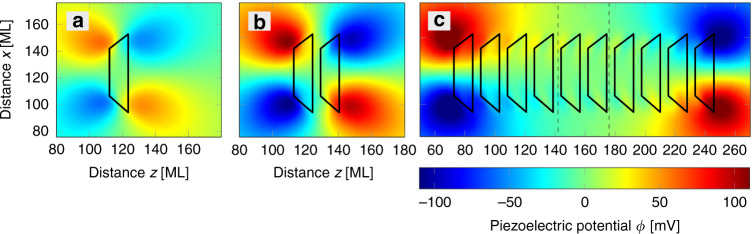
Piezoelectric potential distribution in the (010)-plane cut 20 ML outside of **a** one, **b** two, and **c** stack of ten InAs QDs

Piezoelectric fields accumulate at the first and last QD positions of a stack of vertically aligned QDs and tend to cancel in the center area. Figure 2 depicts the increase in the first and second-order piezoelectric potential with increasing number of QDs﻿ (a) for one QD, (b)﻿ a pair of two QDs, and (c)﻿ a batch of ten QDs, as it may occur in the context of QD based QCLs. The piezoelectric potential is derived by numerically solving the Poisson equation (using Dirichlet boundary conditions) and then adding it as a mean-field to the envelope-function eight-band **k**·**p** Hamiltonian for each grid cell.

### Eight-band $k \cdot p$ method: one-particle states

Using the 8-band **k**·**p** model in real space and nonperiodic boundary conditions, the energy levels and wavefunctions of bound electron and hole states are computed. Originally, the idea was developed to describe electronic states in bulk materials^30–32^. The theory’s envelope-function variant has been further refined and applied to heterostructures such as quantum wells^33^, quantum wires^34^, and quantum dots^13–15,35–37^. Ref. ^34^ explains the rationale behind our approach for zinc blende heterostructures.

This model allows us to consider the impact of strain, piezoelectricity, valence band (VB) mixing, and conduction (CB)-VB interaction on QDs of any geometry and material composition. The predictions of the 8-band **k**·**p** theory are constrained to the Brillouin zone center’s local vicinity owing to the restricted number of Bloch functions utilized for the wavefunction expansion. However, as previously stated, we compute off-center states using the effective mass model: The envelope function approach is used to generate one-particle states for X and L electron states, and the following equation is solved^37^:

$${\widehat{H}}^{L,X}F\left( r \right) = EF\left( r \right)$$

where *E* and $F\left( r \right)$ denote the eigenenergy and their associated envelope function, and ${\widehat{H}}^{L,X}$ is provided by

$${\widehat{H}}^{L,X} = - \frac{{\hbar ^2}}{2}\nabla \cdot \left( {\frac{1}{{\underline{m}}^\ast(r)}} \right)\nabla + E_c^{L,X}(r) + V_{ext}(r)$$

Here, $E_c^{L,X}\left( r \right)$ is the L or X point’s position-dependent bulk conduction band energy, $V_{ext}\left( r \right)$ denotes the external potential generated by, for example, elastic strain.

The amplitude of the strain-induced band shifts is determined by the material-dependent deformation potentials^38,39^. The resulting energy shift at the CB $\Gamma$ point, and the valleys at the L and X points, then reads^38^:

1 $$E_c^i\left( {\hat k_0,\varepsilon } \right) = E_c^i\left( {\hat k_0} \right) + \Xi _d^itr\left( \varepsilon \right) + \Xi _u^i\left( {\hat k_0 \cdot \varepsilon \hat k_0} \right)$$

with the absolute $\Xi _d^i$ and the uniaxial $\Xi _u^i$ deformation potentials, *i* ∈ {$\Gamma$, X, L}; ε denotes the strain.

Meanwhile, an 8-band **k**·**p** implementation for zinc blende and wurtzite QDs is provided through the nextnano^3^ project^40,41^ and is employed herein discussing (InGa)(AsSb)/GaAs/GaP quantum dots.

### Comparison to the sp^3^s* empirical tight-binding method

In order to benchmark our **k**·**p** model, we set up a state-of-the-art sp^3^s* empirical tight-binding (ETB) model. This model has atomistic resolution but considers only a minimal atomic basis, allowing large systems to be computed with high precision in a reasonable time. Similar to **k**·**p**, ETB was developed in the past^42–45^ for full-Brillouin zone description of bulk electronic structure. In ETB, the Schrödinger equation is solved, similarly as for **k**·**p**, and ETB wavefunctions are also envelopes, yet in a basis of atomic orbitals or crystal Bloch waves. Generally, the larger number of basis atomic orbitals is used, the larger part of **k**-space is reproduced by ETB correctly. The usually used bases are sp^3^s*^42^ and sp^3^d^5^s*^43^. The ETB Hamiltonian $\hat H^{ETB}$ is composed of diagonal so-called “on-site” terms related to orbital atomic eigenenergies and off-diagonal ones, called “hopping” terms, related to the interaction between the atomic orbitals of the atom under consideration with its atomic neighbors.

Again, increasing the distance to which the hopping interaction is considered, i.e., the number of neighbors, increases the precision of ETB. Usually, nearest neighbors (NN) or next-nearest neighbors are considered. Since we use an ETB method with orthogonalized atomistic orbitals, it is enough to take into account only interaction with NNs.

The general pattern of constructing Hamiltonian in ETB and **k·p** methods is somewhat similar: diagonal matrix terms include atomic (for ETB) or Bloch wave (for **k·p**) eigenenergies and off-diagonal matrix terms are composed of interactions between atomic orbitals (for ETB) or Bloch states (for **k·p**). Unlike envelope-function **k·p** theory, which computes the electronic states of electrons and holes in a 3D quantum well, in ETB those are interwoven with states of bulk. That complicates their identification, e.g., for nanostructures, as those are “inner” eigenstates of the ETB Hamiltonian and not the ground state.

In the model used, spin–orbit interaction, piezoelectricity and strain are also considered. While piezoelectricity or external electric fields are accounted for in ETB similarly as in **k·p** described earlier, elastic strain is calculated at the atomistic level using the molecular dynamics simulator LAMMPS^43^ and nonlinear Stillinger–Webber potentials of the VFF model^46^. The resulting differences to the unstrained atom positions are realized in ETB model by the direction cosines in the Slater-Koster formulas (angle change) and Harrison’s power law (distance change). For the calculation of the highest occupied and lowest unoccupied states of the InAs QD, calculations at the gamma point and with a sp^3^s* basis per atom provide accurate results. In order to obtain eigenenergies and eigenfunctions we diagonalized ETB matrix using the PETSc/SLEPc numerical package^47,48^. Further details of the model used can be found in the Supplementary information and refs. ^49–55^.

**Fig. 3:**
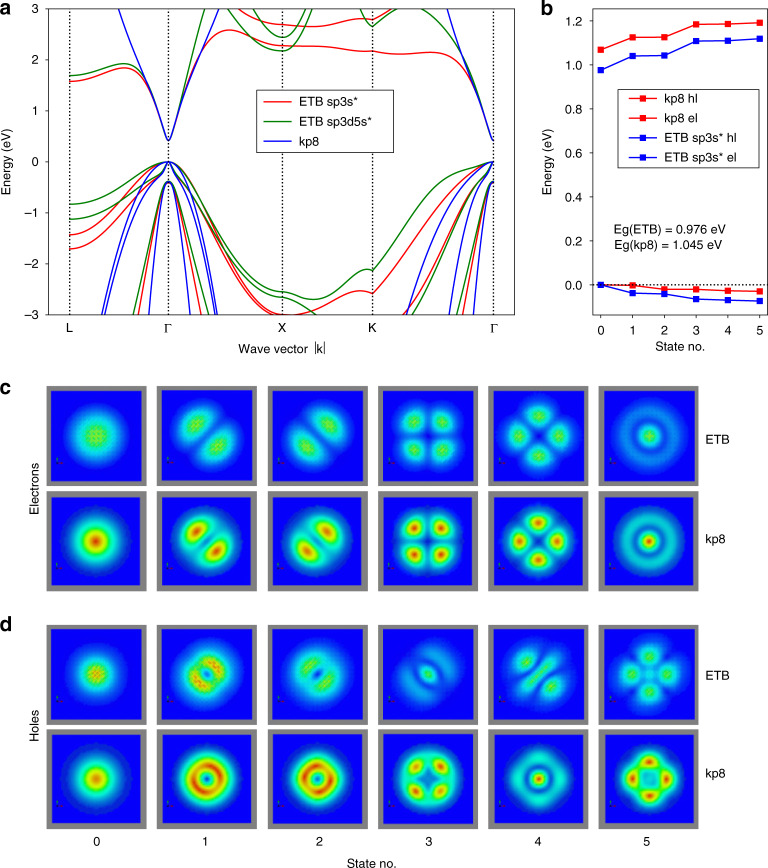
Comparison of the results obtained by eight-band **k·p** (kp8) and sp^3^s* empirical tight-binding (ETB) methods for electronic states of truncated pyramid InAs QD in GaAs matrix with height, basis dimension, and InAs wetting layer thickness of 2.2 nm, 20 nm, and 0.5 nm, respectively. In **a**, we show the band structure of InAs computed using the sp^3^s*, sp^3^d^5^s* ETB, and 8-band **k·p** method. In **b**, we give the eigenenergies of the six states lowest in energy computed by **k·p** and ETB for electrons (el) and holes (hl), respectively. The lateral cuts, taken 1 nm above the base of QD, of the corresponding probability densities are shown in **c** for electrons and **d** for holes. Note that electrons and holes are fully confined inside QD for both **k·p** and ETB. Notice the apparent similarity between ETB and **k·p** results, particularly for the lowest states

We compare the results of envelope-function eight-band **k**·**p** and ETB for a typical InAs QD in GaAs matrix with a 0.5 nm InAs wetting layer, see Fig. 3. The **k**·**p** computations were performed using Nextnano simulation suite^40^, including the calculation of elastic strain by CM model.

The parameters for **k·p** are from the library of Nextnano^40^ and the orthogonal ETB parameters are taken from refs. ^49,50^. For simplicity, piezoelectricity was not considered for calculations in Fig. 3.

One can clearly deduce from Fig. 3 that the results obtained using ETB and eight-band **k·p** are very similar. The differences observed there are mainly due to the dot shapes not exactly matching each other between those methods.

Furthermore, given that sp^3^s* ETB (i) suffers from an inherent ambiguity^56^ of onsite elements at heterostructure interfaces necessitating to use the virtual crystal approximation to restore matrix Hermiticity, (ii) fails to correctly reproduce energies and effective masses^43^ at Brillouin zone edges (see Fig. 3a), and (iii) is computationally much more expensive than eight-band **k·p** (i.e., ~20 h@16 CPU vs. ~1 h@1CPU computation time on a standard computer for ETB and **k·p**, respectively), we find the former not to provide significant advantages over the eight-band **k·p**. The only advantage of the ETB is that it preserves the QD’s correct c_2v_ symmetry of the underlying zincblende lattice owing to its atomistic nature.

### Coupled quantum dots and larger systems

**Fig. 4:**
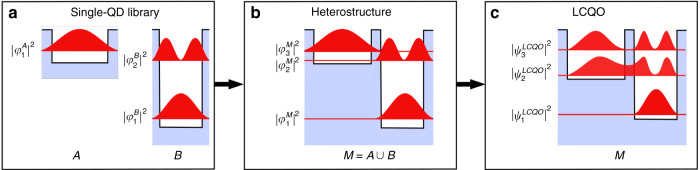
The LCQO-method is illustrated schematically for a two-QD system. **a** A library of basis functions for various sorts of QD is generated. **b** The heterostructure is built using QDs from the library as building blocks, with the subsystems partially overlaid. The Hamiltonian of the system is constructed with strain and piezoelectric potentials taken into account. **c** The heterostructure’s eigenstates are expanded in the union of the basis (reproduced according to ref. ^57^)

To make simulations of large systems with twenty and more electronically coupled QDs more economical, we introduce a new “linear combination of atomic orbitals” type of approximation. Our derivation, the LCQO approach operates along the following principle (See Fig. 4): First, a library of QDs is developed, methodically spanning diverse sizes, shapes, and chemical composition profiles, as well as the related one-particle states. Subsequently, motivated by the specified target properties, arrangements of vertically aligned QDs are built, together with the related Hamilton operator. The latter is then expanded into the basis of one-QD eigenstates as previously stored in our library.

**Fig. 5:**
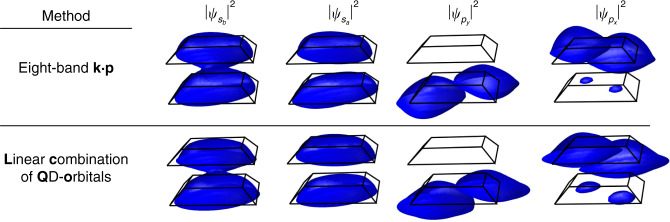
For a couple of two identical InAs QDs (base diameter (along the diagonal): 20.8 nm, height: 2.8 nm, barrier width: 16 MLs), probability densities of the first four-electron states (isosurface at 90%) are shown for the eight-band **k·p** model and the LCQO approach, respectively, with binding and antibinding s-type orbitals, followed by p-type orbitals, respectively (reproduced according to ref. ^57^)

The approach described herein employs basis functions derived using an eight-band **k**·**p** model with realistic structures, strain, and strain-induced internal potentials. Compared to a complete eight-band **k**·**p** calculation for a couple of two QDs, the resulting LCQO eigenstates and -energies exhibit good agreement, as shown in Fig. 5, indicating that the LCQO approach offers reliable results equivalent to those obtained from a full-scale **k**·**p** simulation.

Using the LCQO technique, we can account for the unique 3D morphology of each QD inside the stack, as well as strain and strain-induced internal potentials of the entire system together with inter-dot electronic coupling. The method is introduced and further outlined in Mittelstädt et al.^57^ and can be implemented in conjunction with any continuum or atomistic model employed to determine single-particle states.

Benchmarking for an exemplary system of twenty one-particle states in a series of ten QDs demonstrates that the LCQO technique reduces computing time by at least three orders of magnitude.

## Discussion of selected topics

### Submonolayer QD systems: antimony-driven strong charge-carrier localization

Quantum physics and engineering of III-Sb materials and devices are currently flourishing worldwide. With the development of completely new applications, III-Sb will facilitate volume production of consumer products with improved performance and functionality, such as terahertz transceivers for future 5G mobile systems or low-voltage, low-energy, non-volatile “universal” memories. Energy harvesting green technologies may benefit from the developments of III-Sb-based multi-junction solar cells surpassing the 50% efficiency barrier. Beyond “classical” CMOS, information and communication technologies are now entering the quantum era. Here too, the intriguing electronic and magnetic properties of III-Sb are playing a fundamental role in infrared quantum light sources, spin–photon interfaces, and other quantum communication and information processing devices.

Submonolayer systems are an excellent illustration of how antimony addition may aid to tune the electronic structure and shift emission to longer wavelengths: The cyclic deposition of tiny islands of InAs onto a GaAs matrix results in the development of a customized rough quantum well with densely spaced Indium-rich agglomerations that promote efficient exciton creation.

Antimony alloying improves carrier localization even further, since even modest quantities of Sb incorporation have a huge impact on the arrangement of conduction and valence band positions. Submonolayer QDs exhibit QD-like ultrafast carrier dynamics while also having a much higher modal gain, approaching values seen in InGaAs quantum-well structures.

The extent of hole localization, which is greatly enhanced by the addition of antimony, is the primary difference between alloyed and unalloyed submonolayer quantum dots. As a result, the alloyed submonolayer quantum dots may be used to provide heterodimensional confinement, ranging from totally zero- to hetero-dimensional confinement with completely confined holes and electrons unbound in two dimensions.

**Fig. 6:**
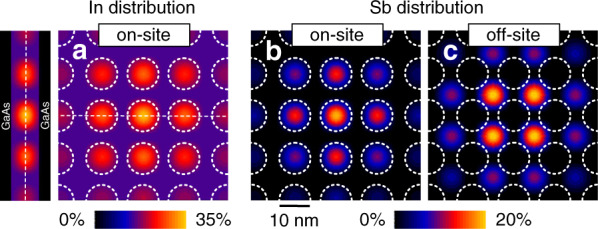
For the 8-band **k·p** calculations, antimony and indium distributions were assumed as follows: The dashed white circles indicate the placement of the indium rich regions, **a** A sigmoidal indium distribution is adopted, with a peak local indium content of 35%. **b** Antimony distributions for scenarios (A) and (C), where the peal local antimony concentration is 20%. (reproduced from ref. ^58^)
B. Antimony is distributed evenly within the SML structure (not displayed)
C. Antimony deposition between the quantum dots (Fig. 6c).

Adding antimony during growth can affect the carrier confinement InAs SML QDs. The influence of Sb alloying on electron and hole wavefunctions was studied employing multi-band **k·p** simulations^58^ for three distinct Sb inclusion arrangements:

A. Antimony incorporation into the quantum dots (Fig. 6b)

Based on earlier data from structural analysis^58^, the simulation’s basic structure is composed of an 8.5 nm thick InGaAs QW with 15% Indium content. Embedded into this QW are circular In-rich accumulations with a cos^2^ composition profile, a peak local indium content of 35%, and a separation of 13.6 nm.

To begin with, introducing antimony into the SML stack results in a shift to lower energies of the PL emission in all three considered scenarios, with the largest shift being 140 meV in case (B). To generate a comparable redshift in the other two situations, a maximum Sb content of higher than 20% is required.

**Fig. 7:**
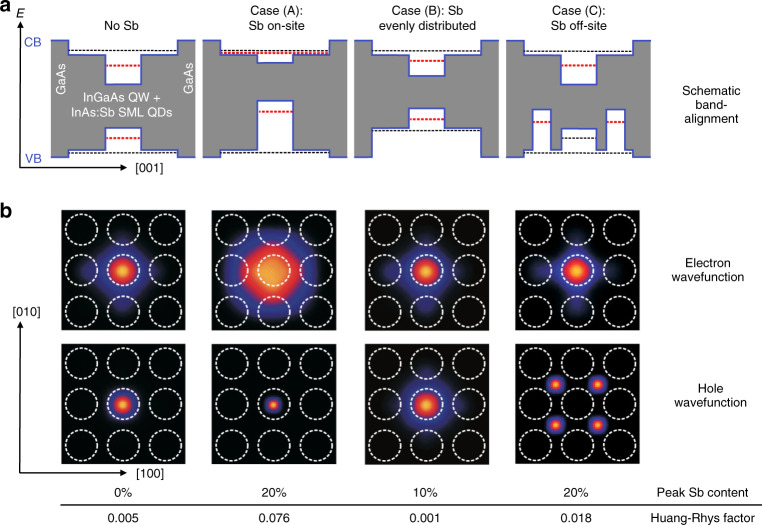
**a** Band alignment diagram for various antimony distribution instances; dotted lines represent the energy levels of the QW and embedded QDs. **b** Localization of electrons and holes for varied Sb distributions. For electron–hole pairs, the Huang-Rhys factors are listed below. (reproduced from ref. ^58^)

As the band alignment scheme in Fig. 7 shows, electron and hole wavefunctions behave highly different when it comes to antimony incorporation: Hole states localize in antimony-rich areas, whereas electron states prefer indium-rich regions and delocalize from antimony-rich regions (Fig. 7b).

The electric dipole moment in the semiconductor structure is influenced by the spatial distribution of the wavefunctions; their overlap defines the Huang-Rhys factor^59^, which is also a metric of LO phonon coupling. A smaller Huang-Rhys factor results from better overlap of the wavefunctions. Figure 7a depicts the band lineup for the three alternative Sb incorporation assumptions, as well as the case without Sb.

In case (A), the incorporation of antimony into QDs causes the hole wavefunction to become more localized, whereas the electron wavefunction delocalizes (Fig. 7a, b). This causes a significant reduction in electron–hole overlap, resulting in a 15-fold rise in the Huang-Rhys factor from 0.005 without Sb to 0.076 at a peak local Sb level of 20%.

An equal distribution of antimony across the SML structure, as in case (B), results in somewhat larger delocalization of the hole wavefunction (Fig. 7b), resulting in stronger overlap and, as a result, a lower Huang-Rhys factor of only 0.001 for 10% antimony content. Case (C), in which antimony is inserted between the QDs, causes the hole wavefunction to be localized at the antimony-rich areas (Fig. 7b), resulting in a substantial delocalization from the QDs; as a result of the decreased overlap, the Huang-Rhys factor increases to 0.018 for 20% Sb content. This scenario corresponds to the smallest total strain in the SML nanostructure.

A comprehensive examination of the PL spectra in ref. ^58^ reveals a coupling strength indicating case (A) to be the most plausible, i.e., a preferred incorporation of antimony in the SML QDs, as well as a shift to lower emission energy for different cases of Sb incorporation in or beside In-rich regions and hole localization at Sb-rich regions. The structural analysis findings also demonstrate that antimony is located in or immediately at the sidewalls of the indium-rich agglomerations (ref. ^58^), resulting in situations similar to scenario (A).

### (InGa)(AsSb)/GaAs/GaP—*k*-indirect transitions

Future information technologies will have to rely on dedicated components that make full use of the quantum technologies. While QDs from III–V compounds were identified as being nearly ideal in that sense, complications arise with integrating those with current mature Si technology^10^. One of the main problems is the sizeable lattice offset between QD semiconductor materials and Si. While that issue can be overcome by gradually growing almost lattice-matched compounds on Si, that approach is technologically complicated and tedious (i.e., necessity of elaborate growth protocols). Thus, it would be favorable to find a semiconductor material with lattice constant close to that of Si. Fortunately, from III–V binary compounds that property is fulfilled by GaP since its lattice is mismatched to that of Si by only 0.37% at 300 K. Sadly, since GaP has the lowest energy transition between conduction and valence band indirect in **k**-space, it cannot be considered useful for construction of light sources. However, it can be used as buffer for growth of other, more appropriate material combinations. First, InGaP alloy was tested in the past for usage as an active material, but it failed due to its type-I to type-II band offset to GaP^60^. On the other hand, (In, Ga)As/GaP shows type-I confinement and was also studied considerably using theory and experimental methods^61–67^. However, that alloy still has a large lattice mismatch to GaP, leading to huge built-in strain, possibly causing a crossover of the ground-state transition from **k**-direct to **k**-indirect in (In, Ga)As/GaP. The necessary fraction of In leading to type-I direct electron-hole transition in (InGa)(AsN)/GaP was found by Fukami et al.^68^ using model-solid theory.

Later on, Robert et al.^69–71^ predicted a change of **k**-direct to **k**-indirect transition for ~30% In concentration for bigger (In, Ga)As QDs in GaP using a mixed **k**·**p**/ETB theory. Interestingly, for smaller QDs they identified an even larger In content for that transition.

Triggered by the experimental works of Sala et al.^72,73^, we^74^ researched the role of further antimony incorporation, i.e., we studied the electronic structure of In_1−*x*_Ga_*x*_As_*y*_Sb_1−*y*_/GaP QDs. We have looked not only at its appropriateness for optoelectronic materials^75^ but also as material for QD-Flash memories that combine long retention time with fast read/write capability, as previously proposed by Sala et al.^73^. Moreover, we note that a fair amount of theory work on Sb containing InAs QD structures was done in the past^76–82^ as well as for QDs with k-indirect transitions^83^ and the study of the present system is a natural expansion of that.

Since GaP substrate causes huge compressive strain in our QDs, in the In_1−*x*_Ga_*x*_As_*y*_Sb_1−*y*_/GaP system the **k**-indirect electron states are found at smaller energies than those for **Γ**. That is in striking difference to more traditionally used QD systems like, e.g., (In, Ga)As QDs in GaAs. Furthermore, because the L (X) bulk Bloch waves have eight- (six-) fold symmetry, there are four (three-) L (X) envelope functions of quasiparticles in this QD system, each state related to two neighboring Brillouin zones. Hence, those envelope wave functions are called L_[110]_, L_[-1-10]_, L_[1-10]_, and L_[-110]_ (X_[100]_, X_[010]_, and X_[001]_).

**Fig. 8:**
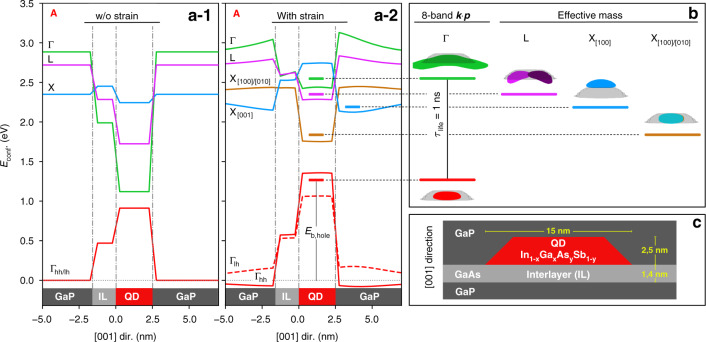
**a** This shows unstrained (a-1) and strained (a-2) energies of *Γ*, L, X_[001]_, X_[100]/[010]_ bands for electrons and holes in QD with Ga and As content being both equal to 20%. The ground-state energies are shown by thick horizontal curves and correspond to side cuts of the envelope functions’ probability densities given in **b**. The modeled QD was truncated cone, with height and base (top) diameter of 2.5 nm, and 15 nm (8 nm), respectively. Below QDs we considered a interlayer of pure GaAs with thickness of 5 ML. **c** The whole structure was finally defined in surrounding GaP matrix

When Eq. (1) is evaluated along QD symmetry-line (and for negligible shear strain, i.e., $\varepsilon _{xy} = \varepsilon _{xz} = \varepsilon _{yz} = 0$), one finds that

$$\begin{array}{lll}E_c^\Gamma \left( {\left[ {000} \right],\varepsilon } \right) &=& E_c^\Gamma + a_c^\Gamma tr\left( \varepsilon \right)\\E_c^L\left( {\left[ {111} \right],\varepsilon } \right) &= &E_c^L + a_c^Ltr\left( \varepsilon \right) + \frac{1}{3}a_{cu}^L\left( {\varepsilon _{xx} + \varepsilon _{yy} + \varepsilon _{zz}} \right)\\E_c^X\left( {\left[ {100} \right],\varepsilon } \right) &=& E_c^X + a_c^Xtr\left( \varepsilon \right) + a_{cu}^X\left( {\varepsilon _{xx}} \right)\\ E_c^X\left( {\left[ {010} \right],\varepsilon } \right) &= &E_c^X + a_c^Xtr\left( \varepsilon \right) + a_{cu}^X\left( {\varepsilon _{yy}} \right)\\ E_c^X\left( {\left[ {001} \right],\varepsilon } \right) &=& E_c^X + a_c^Xtr\left( \varepsilon \right) + a_{cu}^X\left( {\varepsilon _{zz}} \right)\end{array}$$

where $a_c$ and $a_{cu}$ are the absolute deformation potential and the uniaxial shear deformation potential in [100]-direction of the conduction band, respectively. While the term for $E_c^L\left( {\left[ {111} \right],\varepsilon } \right)$ is the same for all L-points, a splitting occurs between $E_c^X\left( {\left[ {100} \right],\varepsilon } \right)$, $E_c^X\left( {\left[ {010} \right],\varepsilon } \right)$, and $E_c^X\left( {\left[ {001} \right],\varepsilon } \right)$. However, for QD’s symmetry-line, $\varepsilon _{xx} = \varepsilon _{yy}$ and, thus, $E_c^X\left( {\left[ {100} \right],\varepsilon } \right)$, $E_c^X\left( {\left[ {010} \right],\varepsilon } \right)$ are identical (see Fig. 8).

**Fig. 9:**
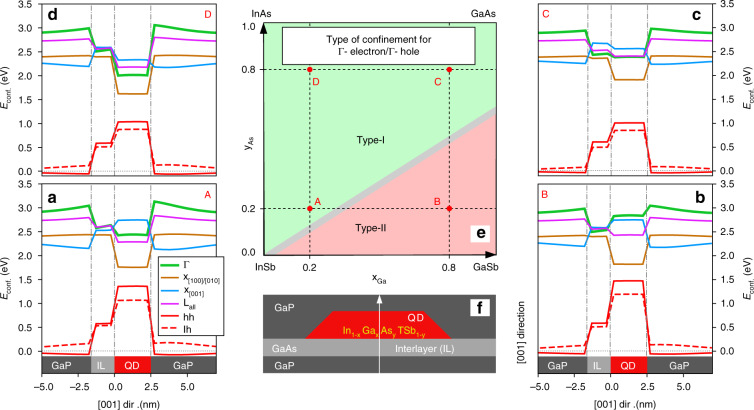
**a**–**e** Local band-edges (**Γ**, X, and L) along the QD vertical symmetry axis [001] depicted by the arrow in **f**. The letters A, B, C, and D represent the material content as shown in **e**. Type of confinement derived from **a**–**e** for **Γ**-electron/**Γ**-hole bands

However, in real In_1−*x*_Ga_*x*_As_*y*_Sb_1−*y*_/GaP QDs, the degeneracy of envelopes for L_[110]_, L_[-1-10]_, L_[1-10]_, and L_[-110]_, or X_[100]_, X_[010]_, and X_[001]_ is canceled because of structural asymmetries (e.g., shape, composition) and/or due to externally applied perturbations (e.g., strain, magnetic, or electric). That effect of degeneracy lifting is studied in Fig. 9 for carefully chosen exemplary points, A, B, C, and D, exhibiting specific properties.

The shape and size of our modeled QDs are related to that found by Stracke and Sala^72,73^ and was recently confirmed by cross-sectional scanning tunneling microscopy (XSTM) and atom probe tomography (APT) measurements^84^, see Fig. 9(f) for the whole structure with 5-ML GaAs interlayer (IL) between QD and GaP substrate.

The local band-edges, computed without and with considering the elastic strain, are shown in Fig. 8a, revealing the vast impact strain has on our system’s confinement properties: In all cases (see also ref. ^74^), we observe a **k**-direct to **k**-indirect transition of the QD-confinement. Antimony incorporation further diversifies the picture, and a real-space type-I to type-II transition for **Γ**-states emerges (Fig. 9e), being a perfect pre-requisite for the QD-Flash memory concept. Furthermore, we note that our model is applicable also in strong and weak confinement regime.

We finally note that the results of our calculations were recently confirmed using detailed power-, temperature-, and time-resolved photoluminescence measurements^75,85^.

### QD based quantum cascade lasers and temperature stability

QCLs are unipolar laser devices wherein electrons run down a staircase potential staircase built from a quantum well (QW) superlattice, and lasing is achieved via intraband transitions. A concept proposed already in 1971 by Kazarinov and Suris^86^.

Despite remarkable advances in material research that resulted in temperature records reported by Bosco et al.^87^ and more recently by Khalatpour et al. in ref. ^88^, however, room temperature operation remains unattained.

The operating temperature is limited on the one hand by an increased competition of radiative transitions with nonradiative scattering losses and absorption of free carriers^89–91^, and on the other hand by a challenging injection of carriers into the upper laser level for transitions at energies below the longitudinal optical phonon frequency of the material (less than 20 meV), so-called terahertz (THz) QCLs. In particular, population inversion is degraded by the continuous subbands in 2-D heterostructures, favoring nonradiative scattering of electrons from the upper laser level and the lower laser level’s thermal backfilling. Since the carriers in the conduction band can easily relax into a lower subband by emitting a phonon, these nonradiative decay processes are much faster than the radiative ones^92–94^, resulting in generally high threshold current densities of QCL devices (∼kA cm^−2^), irrespective of the laser’s transition energy.

To overcome these shortcomings, in 1996 Suris developed the idea of using chains of quantum dots as gain material for quantum cascade lasers (QD-QCLs)^12,95^. Such lasers were predicted to benefit from the inherently narrow gain spectrum, reduced electron–phonon relaxation (phonon bottleneck), and free-carrier absorption of QDs, significantly enhancing carrier lifetimes^96–98^ and thus improving temperature stability with significantly reduced threshold current densities^95,99–101^. Moreover, the operating voltages in conventional QCLs are rather high because the tunneling barriers between adjacent QWs need to be small to form a superlattice. By using QD chains, in contrast, interdot barriers can be selected larger, as the wavefunction penetration in the growth direction is much larger than for comparable QW structures. Consequently, the mutual overlap of the wavefunctions of adjacent QDs is larger than for QW structures with identical spacings, allowing the voltage to be smaller, reducing parasitic tunneling.

A further advantage of using QDs is their anisotropic radiation pattern, enabling the design of surface-emitting lasers based on strong vertical emission of s-to-p-like intraband transitions. Then, THz QD-QCLs, in particular, would benefit from a facile coupling into optical fibers, low-cost production, and high output powers by operation in arrays.

#### Proposal for a room-temperature THz laser

**Fig. 10:**
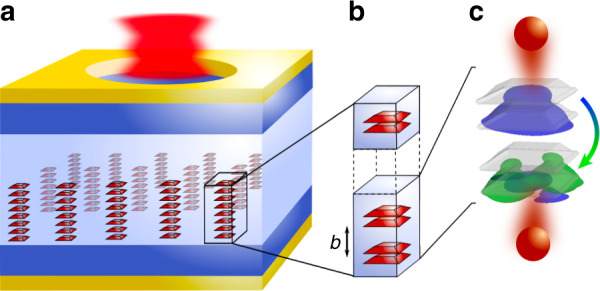
Scheme of a vertical-cavity surface-emitting QD chain-based QCL. **a** Electronically coupled QDs form an array of QD chains serving as the QCL’s gain material. **b** QDs separated by tunneling barriers of width *b* arranged in two-QD unit cells. **c** Vertical spontaneous emission from transitions between s-type (blue) and p-type (green) QD orbitals

We present a QD chain’s band structure calculation featuring an intraband staircase potential with a laser transition in the THz regime. The THz QCL design comprises an array chain built from 20 stacked InGaAs QDs embedded in a GaAs matrix forming a two-QD unit-cell superlattice; see Fig. 10. Delocalized electronic states resulting from strong electronic interdot coupling are tailored to enable occupation inversion at a specific range of external biases. With the help of transport calculations built on the calculated band structures and device parameters mapped from experiments, we find a significantly reduced threshold current density and higher temperature stability compared to QW-based cascade lasers.

The computational cost of finding a suitable QD gain material is less the calculation of (1) the electronic structure of large-scale finite QD systems, but scales primarily with the computation of (2) dozens of electronic states of these QD chains and the (3) numerous iterations required to arrive at a suitable bandstructure design. The latter are, therefore, the major challenges in a design study. Thereby, the morphology, composition, length of the QD chain, and width of the tunneling barriers of the QDs along the QD chain, which affects the interdot coupling strength, were varied to arrive at a promising band structure. The realization of a QCL requires a meticulous band structure design. In particular, this requires, on the one hand, cascaded intraband transitions of matched energy and shared carrier probability densities of neighboring QDs and, on the other hand, in- and out-scattering rates allowing population inversion between the laser levels. In an extensive parameter study, using the results and discussions in ref. ^57^ and our newly developed LCQO method, we have investigated more than one hundred possible realizations of QD chains within experimentally realistic parameters at various external biases. The QD-QCL active region presented consists of In_0.7_Ga_0.3_As QDs electronically coupled via tunneling barriers of width *b* and modeled as truncated pyramids, following transmission electron microscopy investigations of Stranski-Krastanov QDs^102,103^.

Our design uses a two-QD unit cell with QDs coupled across an 8 ML barrier, where barriers of 20 MLs separate neighboring unit cells. Consequently, the densities within the two-QD unit-cells are well localized and the overlap of carrier densities from cascade to cascade is reduced accordingly, minimizing the leakage current from the upper laser level and facilitating population inversion. The band structure is motivated by designs already used in QW-based QCLs that attained record temperatures; cf. refs. ^87,91,104,105^.

**Fig. 11:**
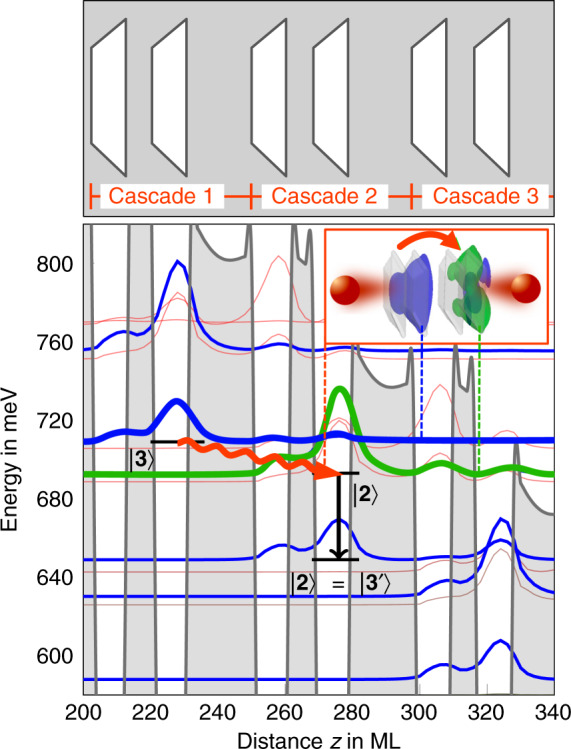
Segment of the conduction band staircase potential for the QD chain of 20 InGaAs/GaAs QDs (shaded gray), with an alternating barrier sequence of 8 and 20 monolayers. The single-particle states of the QD chain are depicted in blue, with the upper laser level $\left| 3 \right\rangle$ (s-type) and the lower laser level $\left| 2 \right\rangle$ (px, y-type). The wavy red arrow indicates the laser transition, and the orbitals involved are displayed in the inset. Shown are two cascades of the QD chain, which consists of seven in total. The applied external bias voltage is set to 46 kV/cm

Figure 11 shows a segment of the QD chain’s band structure, resulting in seven cascades along the 20 QD chain. There are seven laser transitions between the unit cells and four QDs at the top and bottom; cf. the Supplemental Material of ref. ^57^. The arrows indicate the laser transition between adjacent two-QD unit cells and the depletion of the lower laser level via nonradiative transitions within the unit cell. Although only these two transitions are indicated for the sake of clarity, in our transport model^106^, we consider such radiative and nonradiative transitions and the reverse processes for each state along the chain, resulting in 35 coupled rate equations.

We note that the laser transition is engineered between the conduction band ground state (s-type symmetry) of a unit cell and the subsequent unit cell’s excited state (p-type symmetry).

Since the lower laser level is an excited QD state, the nonradiative scattering out of a unit cell’s p-type state into the s-type ground state, as well as into the wetting layer and bulk, is much more efficient than for the upper laser level, which is a ground state. Hence, population inversion is maintained for a specific externally applied bias range as long as the scattering from the upper laser level is less efficient than from the lower laser level into the two-QD unit cell’s ground state and the thermal backfilling of the lower laser level.

The staircase structure facilitates population inversion analogous to the reduced QW-based THz QCL active region designs, where the ground state serves as the subsequent cascade’s upper laser level, omitting an additional injector region^87,107–109^, maximizing the number of cascades and thus the gain of the active material. These reduced designs also feature a diagonal laser transition, as shown in Fig. 11, allowing the laser transition’s energy to be continuously tuned from ∼8 to 17 meV with an external bias of 42–48 kV/cm. In contrast to QW-based QCL designs, the QD-laser transition has no restrictions with respect to emission directionality. Hence, as reported in ref. ^106^, both top- and edge-emitter designs are feasible using quantum dot chains as active QCL material.

## Conclusions

In this article, we provided an overview on the envelope-function-based eight-band **k**·**p** method and the newly developed “linear combination of quantum dot orbitals” method for electronic structure calculations of small and large systems of QDs. Furthermore, we have benchmarked our **k·p** method against an atomistic sp^3^s* empirical tight-binding algorithm, which we have set up, finding it a fast and reliable alternative to its atomistic counterpart.

Furthermore, three types of heterostructures of current relevance are utilized to demonstrate the **k·p** approaches’ broad range of application:

(i) the electronic and optical properties of InAs/GaAs submonolayer stacks with Sb as an extra atomic species—enabling significant shifts of the emission to longer wavelengths,
(ii) In_1−*x*_Ga_*x*_As_*y*_Sb_1−*y*_ lattice-matched to GaP, thus, possibly paving the path to monolithic integration of III–V compounds with Si technology, and
(iii) the application of our newly developed LCQO method and its application to the design of a terahertz QD-QCL, aiming at lasing at room temperature. The LCQO method enables and accelerates the computation of the band structure of QD stacks QD stacks with 20 or more QDs, including dozens of electronic states, at an affordable computational cost while maintaining the accuracy provided by the eight-band **k·p** method.

## Supplementary information

Supplementary Information
