# Supplementary material for: Modeling electronic and optical properties of III–V quantum dots—selected recent developments
Source: Light Sci Appl. 2022 Jan 17;11:17. doi: 10.1038/s41377-021-00700-9 (PMC8761749; doi:10.1038/s41377-021-00700-9)
Supplement: Supplementary file 1 — Supplementary Information [file 41377_2021_700_MOESM1_ESM.pdf]

Supplementary Information for Modeling electronic and optical properties of III-V quantum dots – selected recent developments

Alexander Mittelstädt,[1] Andrei Schliwa,[1] and Petr Klenovský[2, 3, *]

[1] *Institute for Solid State Physics, Technical University of Berlin, Hardenbergstrasse 36, D-10623 Berlin, Germany*
[2] *Department of Condensed Matter Physics, Faculty of Science, Masaryk University, Kotlářská 267/2, 61137 Brno, Czech Republic*
[3] *Czech Metrology Institute, Okružní 31, 63800 Brno, Czech Republic*

I. EMPIRICAL TIGHT BINDING MATRICES FOR III-V SEMICONDUCTORS

Here we provide examples of the Empirical tight binding (ETB) matrices used in our work together with an outline of the employed ETB method. The general theory with all its bifurcations is discussed in length within Refs. 1–3.

Following Ref. 2 and 3, the stationary Schrödinger equation for bulk semiconductor with Löwdin-orthogonalized states, which needs to be solved is the following

$$\sum_{\alpha'\nu'\sigma'} \widehat{H}^{\mathrm{bulk}}_{\alpha\nu\sigma,\alpha'\nu'\sigma'} u_{\alpha'\nu'\sigma'} = E(\mathbf{k}) u_{\alpha\nu\sigma}, \tag{1}$$

where α marks the atom type, ν the atomic orbital, and σ the spin. The function $u_{\alpha\nu\sigma}$ denotes the Bloch factor and $\mathbf{k}$ the reciprocal lattice vector. Furthermore,

$$\widehat{H}^{\mathrm{bulk}}_{\alpha\nu\sigma,\alpha'\nu'\sigma'} = \sum_j e^{\mathrm{i}\mathbf{k}\mathbf{R}_j} \langle \mathbf{0}\alpha'\nu'\sigma'| \widehat{H}^{\mathrm{bulk}} |\mathbf{R}_j\alpha\nu\sigma\rangle \tag{2}$$

where j enumerates the neighbors and $\mathbf{R}_j$ marks the relative vector from atom to its j-th neighbor. The bracket on right hand side denotes the parameters which are fitted to the bandstructure, thus, justifying the prefix "empirical" to the method.

In this work we consider the basis of atomic orbitals $\nu \in \{s, p_x, p_y, p_z, s^*\}$ where the orbital s^* is an effective orbital collating the effects of d atomic orbitals [4] and we will call that basis in shorthand notation sp^3s^*. The matrix in Eq. (2) consist of terms where $\mathbf{R}_j = 0$, i.e., so-called "on-site" terms $\widehat{O}_{\alpha\alpha}$ referring to energies of the bare atomic orbitals and "hopping" or "off-site" terms $\widehat{I}_{\alpha\beta}$ for $\mathbf{R}_j \neq 0$, describing the interaction of atom $\alpha \in \{a, c\}$, where a and c denotes anion and cation, respectively, with its neighboring atoms $\beta \in \{c, a\}$. Note, that the order is reversed for β since in this work only nearest neighbors (NN) are considered and the neighbor of anion in zincblende crystal is always cation and vice versa.

Furthermore, we deal in this work with quantum dots (QDs) of direct band semiconductors, i.e., $\mathbf{k} = 0$ in our case. Thus, the ETB Hamiltonian for QD then has the form

$$\widehat{H}^{\mathrm{QD}} = \sum_{\mathbf{0}\alpha\nu\sigma} E_{\alpha\nu} |\mathbf{0}\alpha\nu\sigma\rangle \langle\mathbf{0}\alpha\nu\sigma| + \sum_{\mathbf{0}\alpha\nu\sigma,\mathbf{R}'\alpha'\nu'\sigma'} V_{\mathbf{R}'\alpha'\nu'\sigma'\mathbf{0}\alpha\nu\sigma} |\mathbf{R}'\alpha'\nu'\sigma'\rangle \langle\mathbf{0}\alpha\nu\sigma|, \tag{3}$$

where $\mathbf{0}$ marks the position of each considered atom and $\mathbf{R}'$ the relative position of NN with respect to atom at $\mathbf{0}$.

The block matrices from which the Hamiltonian of ETB with sp^3s^* atom orbital basis is constructed, have the size 5×5. For on-site elements they read

$$\widehat{H}_{\alpha\alpha} = \begin{pmatrix} E_s^\alpha & & & & \\ & E_p^\alpha & & & \\ & & E_p^\alpha & & \\ & & & E_p^\alpha & \\ & & & & E_{s^*}^\alpha \end{pmatrix}, \tag{4}$$

and involve three independent elements E_s^α, E_p^α, and $E_{s^*}^\alpha$, for each of the considered atomic orbital and a given atom species, i.e., six in total for binary zincblende semiconductors.

* klenovsky@physics.muni.cz

On the other hand, the hopping matrices for NN approximation are

$$\widehat{H}_{ac}^{\rm nn} = \begin{pmatrix} V_{ss\sigma} & d_x V_{sp\sigma}^{ca} & d_y V_{sp\sigma}^{ca} & d_z V_{sp\sigma}^{ca} & 0 \\ -d_x V_{ps\sigma}^{ac} & V_{pp\pi} + d_x^2 \widetilde{V} & d_x d_y \widetilde{V} & d_x d_z \widetilde{V} & -d_x V_{ps^*\sigma}^{ac} \\ -d_y V_{ps\sigma}^{ac} & d_x d_y \widetilde{V} & V_{pp\pi} + d_y^2 \widetilde{V} & d_y d_z \widetilde{V} & -d_y V_{ps^*\sigma}^{ac} \\ -d_z V_{ps\sigma}^{ac} & d_x d_z \widetilde{V} & d_y d_z \widetilde{V} & V_{pp\pi} + d_z^2 \widetilde{V} & -d_z V_{ps^*\sigma}^{ac} \\ 0 & d_x V_{s^*p\sigma}^{ca} & d_y V_{s^*p\sigma}^{ca} & d_z V_{s^*p\sigma}^{ca} & 0 \end{pmatrix}, \tag{5}$$

for on-site atom being anion and, thus, its nearest neighbors being cations, and

$$\widehat{H}_{ca}^{\rm nn} = \begin{pmatrix} V_{ss\sigma} & d_x V_{ps\sigma}^{ca} & d_y V_{ps\sigma}^{ca} & d_z V_{ps\sigma}^{ca} & 0 \\ -d_x V_{sp\sigma}^{ac} & V_{pp\pi} + d_x^2 \widetilde{V} & d_x d_y \widetilde{V} & d_x d_z \widetilde{V} & -d_x V_{s^*p\sigma}^{ac} \\ -d_y V_{sp\sigma}^{ac} & d_x d_y \widetilde{V} & V_{pp\pi} + d_y^2 \widetilde{V} & d_y d_z \widetilde{V} & -d_y V_{s^*p\sigma}^{ac} \\ -d_z V_{sp\sigma}^{ac} & d_x d_z \widetilde{V} & d_y d_z \widetilde{V} & V_{pp\pi} + d_z^2 \widetilde{V} & -d_z V_{s^*p\sigma}^{ac} \\ 0 & d_x V_{ps^*\sigma}^{ca} & d_y V_{ps^*\sigma}^{ca} & d_z V_{ps^*\sigma}^{ca} & 0 \end{pmatrix}, \tag{6}$$

for the other possible combination in zincblende semiconductors. The matrices in Eqs. (5) and (6) involve seven more independent elements $V_{ss\sigma}$, $V_{sp\sigma}^{ca}$, $V_{ps\sigma}^{ac}$, $V_{s^*p\sigma}^{ca}$, $V_{ps^*\sigma}^{ac}$, $V_{pp\pi}$, and $\widetilde{V}$. Thus, taking into account also the on-site elements, thirteen independent variables need to be fitted to the bandstructure of each particular semiconductor for sp^3s^* ETB. Furthermore, the following relations hold

$$\widetilde{V} = (V_{pp\pi} - V_{pp\sigma}), \tag{7}$$
$$V_{pp\pi} = (V_{xx} - V_{xy}), \tag{8}$$
$$V_{pp\sigma} = (V_{xx} + 2V_{xy}), \tag{9}$$
$$V_{sp\sigma}^{ac} = \sqrt{3}\, V_{sp}^{ac}, \tag{10}$$
$$V_{ss\sigma} = V_{ss}. \tag{11}$$

The parameters in Eqs. (7)–(11) are related to the Slater-Koster formulas [5] and are multiplied by the directional cosines of the bonds from an atom to its NN, i.e.,

$$d_\mu = \frac{\mathbf{e}_\mu \cdot \mathbf{d}_{ij}}{d_{ij}};\ d_{ij} = |\mathbf{d}_{ij}|;\ \mu \in \{x, y, z\}, \tag{12}$$

and scaled by the Harrison's power law [6]

$$\widehat{H}_{ac}^{\rm QD} = \widehat{H}_{ac}^{nn} \left(\frac{d_{ij}^0}{d_{ij}} \right)^\eta, \tag{13}$$

where d_{ij}^0 and d_{ij} are the distances to NN for unstrained and strained material, respectively, and we have considered $\eta = 2.9$ following Refs. 2 and 3.

To describe also the effect of spin in our single particle ETB calculations, we added that to the ETB Hamiltonian onsite terms:

$$\widehat{O}_{\alpha\alpha} = \begin{pmatrix} \widehat{H}_{\alpha\alpha}^{5\times5} & \\ & \widehat{H}_{\alpha\alpha}^{5\times5} \end{pmatrix} + \lambda \cdot \begin{pmatrix} 0 & 0 & 0 & 0 & 0 & 0 & 0 & 0 & 0 & 0 \\ 0 & 0 & -\mathrm{i} & 0 & 0 & 0 & 0 & 0 & 1 & 0 \\ 0 & \mathrm{i} & 0 & 0 & 0 & 0 & 0 & 0 & -\mathrm{i} & 0 \\ 0 & 0 & 0 & 0 & 0 & 0 & -1 & \mathrm{i} & 0 & 0 \\ 0 & 0 & 0 & 0 & 0 & 0 & 0 & 0 & 0 & 0 \\ 0 & 0 & 0 & 0 & 0 & 0 & 0 & 0 & 0 & 0 \\ 0 & 0 & 0 & -1 & 0 & 0 & 0 & \mathrm{i} & 0 & 0 \\ 0 & 0 & 0 & -\mathrm{i} & 0 & 0 & -\mathrm{i} & 0 & 0 & 0 \\ 0 & 1 & \mathrm{i} & 0 & 0 & 0 & 0 & 0 & 0 & 0 \\ 0 & 0 & 0 & 0 & 0 & 0 & 0 & 0 & 0 & 0 \end{pmatrix}, \tag{14}$$

and the hopping terms need to be changed accordingly

$$\widehat{I}_{ac} = \begin{pmatrix} \widehat{H}_{ac}^{5\times5} & \\ & \widehat{H}_{ac}^{5\times5} \end{pmatrix}. \tag{15}$$

Note that the spin brings another empirical parameters λ which needs to be fitted to the bandstructure, increasing the total number of independent terms to 14.

Furthermore, we give a toy example of ETB Hamiltonian for (few atom) quantum dot (QD) nanostructure to elucidate the construction of the ETB matrix

$$\widehat{H}_{\mathrm{QD}} = \begin{pmatrix} \widehat{O}^{1}_{aa} & & & \widehat{I}^{14}_{ac} & \\ & \widehat{O}^{2}_{cc} & \widehat{I}^{23}_{ca} & & \\ & \widehat{I}^{23}_{ac} & \widehat{O}^{3}_{aa} & & \widehat{I}^{35}_{ac} \\ \widehat{I}^{14}_{ca} & & & \widehat{O}^{4}_{cc} & \\ & & \widehat{I}^{35}_{ca} & & \widehat{O}^{5}_{cc} \end{pmatrix}, \tag{16}$$

where the following interactions to NN are considered:

- atom 1 has one NN atom no. 4,
- atom 2 has one NN atom no. 3,
- atom 3 has two NN atoms no. 2 and 5,
- atom 4 has one NN atom no. 1,
- atom 5 has one NN atom no. 3.

Finally, in Fig. 1 we provide the InAs/GaAs QD atomistic structure, the results of which are shown in Fig. 3 of the main paper body.

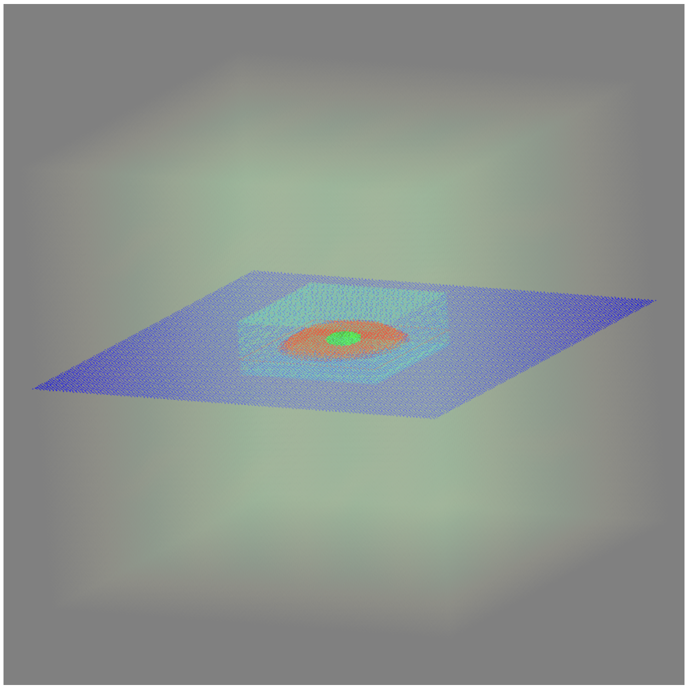

FIG. 1. Simulated InAs/GaAs QD atomistic structure [7] using the sp^3s^* ETB code. The QD is shown in red, the InAs wetting layer in dark blue, the ground state hole probability density in green, and by light blue is marked the space in which the matrix diagonalization using PETSc/SLEPc [8, 9] is performed.

[1] S. Schulz, Ph.D. thesis, Universität Bremen (2007).
[2] E. Goldmann, Ph.D. thesis, Universität Bremen (2014).
[3] C. Carmesin, Ph.D. thesis, Universität Bremen (2018).
[4] P. Vogl, H. P. Hjalmarson, and J. D. Dow, Journal of Physics and Chemistry of Solids **44**, 365 (1983).
[5] J. C. Slater and G. F. Koster, Physical Review **94**, 1498 (1954).

[6] W. A. Harrison, *Electronic Structure and the Properties of Solids*, (Dover Publications, 1989).

[7] LAMMPS molecular dynamics simulator. (2021). at https://www.lammps.org/.

[8] L. D. Dalcin, R. R. Paz, P. A. Kler, and A. Cosimo, Advances in Water Resources **34**, 1124 (2011), new Computational Methods and Software Tools.

[9] J. E. Roman, C. Campos, L. Dalcin, E. Romero, and A. Tomas, *SLEPc Users Manual: Scalable Library for Eigenvalue Problem Computations*, D. Sistemes Informàtics i Computació, Universitat Politècnica de València (2021).
